# Spectra-structure correlations in NIR region of polymers from quantum chemical calculations. The cases of aromatic ring, C=O, C≡N and C-Cl functionalities

**DOI:** 10.1016/j.saa.2021.120085

**Published:** 2021-06-16

**Authors:** Krzysztof B. Beć, Justyna Grabska, Jovan Badzoka, Christian W. Huck

**Affiliations:** Institute of Analytical Chemistry and Radiochemistry, University of Innsbruck, Innrain 80-82, 6020 Innsbruck, Austria

**Keywords:** Near-infrared (NIR) spectroscopy, Quantum chemical calculation, Overtones, Combination bands, Polymers, Microplastic

## Abstract

Near-infrared (NIR) spectroscopy is a valued analytical tool in various applications involving polymers. However, complex nature of NIR spectra imposes difficulties in their direct interpretation. Here, anharmonic quantum chemical calculations are used to simulate NIR spectra of nine polymers; acrylonitrile butadiene styrene (ABS), ethylene–vinyl acetate (EVAC), polycarbonate (PC), polyethylene terephthalate (PET), polylactide or polylactic acid (PLA), polymethylmethacrylate (PMMA), polyoxymethylene (POM), polystyrene (PS) and polyvinylchloride (PVC). The generalized spectra-structure correlations are derived for these systems with focus given to the manifestation in NIR spectra of aromatic ring, C=O, C≡N and C-Cl functionalities. It is concluded that the nature of NIR polymer bands is only moderately sensitive to the remote chemical neighborhood. The majority of NIR absorption of polymers originates from binary combination bands, while the first overtones are meaningful only in ca. 6200-5500 cm^−1^ region. The contribution of the overtone bands is relatively higher for the polymers bearing aromatic rings because of higher intensity of C-H stretching overtones. Highly characteristic combination bands of the modes localized in aromatic ring (ring deformation and CH stretching) are relatively independent on the remaining structure of the polymer. The combination bands originating from C=O group are more sensitive to the chemical neighborhood in near proximity, forming a useful fingerprint for a specific polymer. In contrast, the vibrational bands of C≡N functionality are far less useful in NIR region than in infrared (IR) region. With aid of the calculated absorption bands, structural specificity of NIR spectroscopy of polymers can be markedly improved.

## Graphical abstract

**Graphical Abstract:**
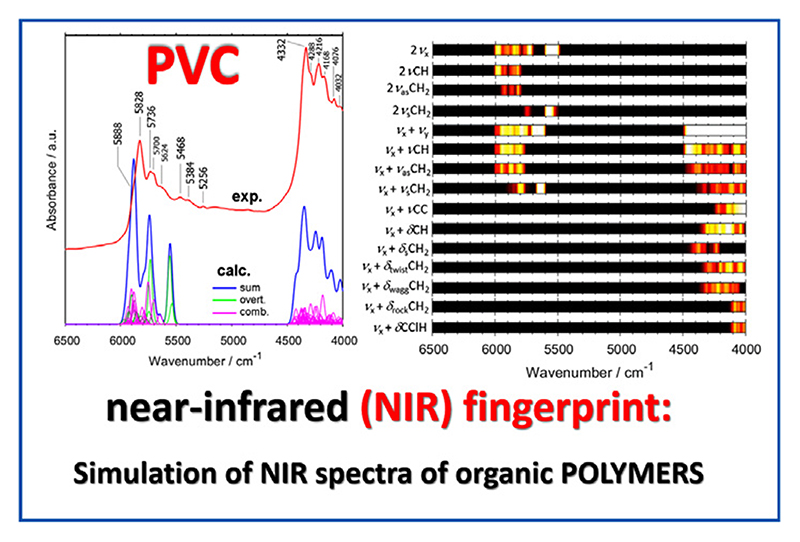

## 1 Introduction

Near-infrared (NIR; 12500–4000 cm^−1^) spectroscopy is a vibrational spectroscopy technique particularly valued in practical applications [1–4]. Compared with mid-infrared (MIR; 4000–400 cm^−1^) spectroscopy [5], this technique offers the ability to obtain good quality spectra from a bulk sample without prior sample preparation, while operating in a non-contact, diffuse reflectance mode. NIR spectroscopy is valued as a non-destructive, cost-effective, fast and efficient tool for qualitative and quantitative analyzes in diverse areas [1], in particular in the analytical applications related to agriculture [6,7], food [8,9], natural products [10] or pharmaceuticals [11] and environmental monitoring as well [12]. It is also a valued tool for the qualitative and quantitative analysis of fuels [13–15] and polymers [16–19] including textiles [20–22].

NIR spectroscopy is also an essential too for basic research given its distinction to other types of vibrational spectroscopy [23,24]. NIR spectra are populated exclusively by non-fundamentals bands, i.e. overtones and combination bands, from which one can elucidate the information on anharmonicity of molecular vibrations, chemical structure, intra- and intermolecular interactions, and solvent interaction. Notwithstanding, NIR spectroscopy also faces significant limitations and challenges related to the complexity of the spectra and difficulties in their direct interpretation [23,24]. In contrast to MIR spectra, the absorption bands observed in NIR region are broad and heterogeneous as the result of an extensive level of band overlapping [25]. The interpretability of MIR or Raman spectra can routinely be assisted by quantum-chemical calculation of vibrational bands based on a straightforward double harmonic approximation [26,27]. However, this efficient approach does not provide information on the overtones and combination bands and thus is not applicable for approximation of NIR spectra [26,27,28]. The harmonic frequencies are only suitable for approximating the experimental positions of the fundamental bands. Overtone bands intensities are equal to zero as the consequence of the linear approximation of the dipole moment, i.e. neglecting electrical anharmonicity. Furthermore, the additive character of the harmonic potential neglects the mode coupling [26].

Given the intrinsic complexity of NIR spectra, the traditional empirical methods used in spectroscopy for interpretation of vibrational bands (e.g. analysis of a series of analogous compounds [29] or isotopic substitution [30,31]) have been largely limited [32]. Furthermore, an attempt to identify the bands of the same origin by establishing correlations between IR and NIR spectra of the same sample [33–35] may be misleading, as the anharmonic shifts and intensity variations are inconsistent between different modes [36,37]. Consequently, the chemical specificity of NIR spectra is inferior to that of MIR or Raman spectra [38]. It also forms a considerable drawback in analytical applications, in which an improved understanding of NIR spectra can result in a better design of analytical approach [39]. Theoretical prediction of NIR spectra requires computationally expensive anharmonic methods [28]. In recent years anharmonic calculations of NIR spectra have become feasible for practically meaningful molecules [28], ranging from e.g. alcohols [40,41], through carboxylic acids [42], phenols [39] to long-chain fatty acids [43]. The insights were gained from these studies into the origins of NIR absorption lineshape, correlations between the spectra and molecular structure, and the effects of the intermolecular interactions and solvent effect as well. However, with few exceptions [44,45], the majority of these studies were aimed at the samples diluted in a relatively inert solvent (i.e. carbon tetrachloride), in order to reduce the uncertainty from not yet fully treatable problem of the chemical neighborhood effects present in neat liquid or solid phase.

In this work attention is given to NIR spectra of organic polymers. These chemicals are important subjects from the point-of-view of the applications of NIR spectroscopy. In addition to wide industrial applications [16,46], in the last few years an increasing focus is directed towards the detection and identification of microplastic pollution by NIR spectroscopy [47–50].Therefore, considerable efforts are being directed at establishing an analytical method suitable for wide-scale detection and identification of the pollutants. Given the advantages of NIR spectroscopy outlined above, this technique is considered to be a promising tool for such role [47,48,49,50,51]. On the other hand, NIR spectroscopy has been used with great success to monitor the polymerization reaction, the kinetics and the composition of the product [46,52]. Despite the importance of polymers, their NIR spectra have never been studied with aid from quantum chemical calculations. Previous investigations of simpler molecules evidenced that the long range chemical neighborhood is relatively less meaningful in NIR spectra in contrast to MIR spectra [44,45], and that a relatively accurate theoretical prediction of NIR spectra is possible even with the use of simplified molecular models, in which the key structural motif of a larger system is included [45]. Therefore, it would be interesting to verify whether a similar approach can be assumed to approximate the NIR spectra of polymers based on simplified models that include only one or few units of a polymer in order to reduce the complexity of the computations to an acceptable level.

To satisfy the aims of this investigation, 9 polymers were studied: acrylonitrile butadiene styrene (ABS), ethylene–vinyl acetate (EVAC), polycarbonate (PC), polyethylene terephthalate (PET), polylactide or polylactic acid (PLA), polymethylmethacrylate (PMMA), polyoxymethylene (POM), polystyrene (PS) and polyvinylchloride (PVC). These systems offer several advantages for the purpose of this work. Firstly, they share broad and essential practical significance in various applications. Hence, they fall into materials routinely analyzed by NIR spectroscopy e.g. in quality control. Detailed characterization of the NIR spectra of the studied polymers will be beneficial for analytical applications of NIR spectroscopy. Furthermore, these polymers feature molecular structures that offer particularly interesting properties to be investigated in their NIR spectra. ABS, PC, PET and PS feature an aromatic ring in their structure. The NIR spectral footprint of the co-existing aromatic rings and aliphatic groups is particularly interesting, as are the combination bands involving these modes. Next, the manifestation of C=O moiety in the NIR spectra of polymers is of great interest given the known distinctiveness of these vibrations reported for small molecules [53]. Insightful should be also the investigation of the C≡N bands in the NIR spectra. Beyond the fundamental spectra-structure correlations, this study aims to shed light on the effects of long-range chemical neighborhood on NIR spectra as well. Finally, the suitability of using simplified molecular models that capture the main structural motifs of the considered larger systems will be further evaluated for polymers to complement previous findings made for melamine and nucleobases [44,45].

## 2 Materials and methods

### 2.1 Experimental

The polymer standards were acquired from the suppliers present at the commercial market (Arkema, INEOS Styrolution, INOVYN, NatureWorks LLC, Rohm GmbH, Saudi Basic Industries Corporation SABIC). NIR spectra were measured on Fourier-transform (FT) Büchi NIRFlex N-500 spectrometer equipped with polarization interferometer and the accessory for measurement of solid samples in diffuse reflectance mode. The spectra were acquired in the region of 10,000 to 4000 cm^−1^ with the spectral resolution of 8 cm^−1^, interpolated to 4 cm^−1^, resulting in 1501 data points per spectrum. For each spectrum a total number of 64 averaged scans were collected. The spectrometer was operated by the software Büchi NIR Ware 1.4.3010.

### 2.2 Quantum chemical calculations of NIR spectra

Computations of NIR spectra were based on anharmonic vibrational analysis performed by means of the Deperturbed Vibrational Second-Order Perturbation Theory (DVPT2) [54,55]. The underlying geometry optimization of the models and the determination of the energy was carried out using DFT approach at B3LYP/6-311+g(df,pd) level of electronic theory, additionally refined by applying Grimme’s third version of empirical correction for dispersion with Becke-Johnson damping (GD3BJ) [56]. The calculations included up to two quanta transitions, i.e. the first overtones and binary combinations; this is sufficient to accurately reconstruct an NIR spectrum [30]. All quantum mechanical calculations were performed with Gaussian 16 Rev. C01 software [57]. The modeling of the spectral lineshape was carried out through parameterized band broadening. Lorentz-Gauss (Cauchy-Gauss) product function was used as the bandshape model [58].

## 3 Results and discussion

### 3.1 Considerations towards model size suitable for anharmonic calculations

**Fig. 1:**
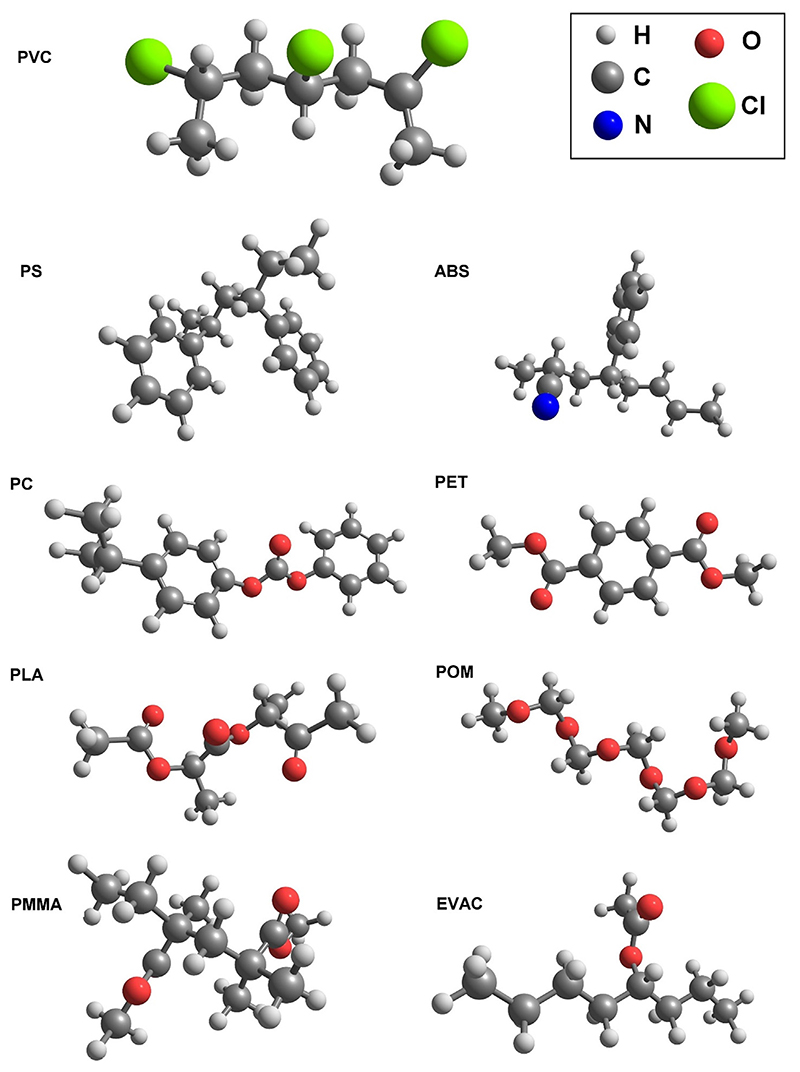
The models representing the structures of the investigated polymers. Refer to SI for further details.

The previous studies indicated that the simplified models, which represent the structural motifs of larger systems (i.e. crystal lattice) can be successfully used to approximate NIR spectra. These conclusions were drawn for melamine and nucleobases and corresponded to most of the out-of-plane vibration in these systems being largely irrelevant for NIR spectra. Therefore, it is of keen interest to verify whether a similar approach can be assumed for organic polymers with different structures and functionalities. In this case, this approach seems feasible for different reason. NIR spectra are populated mostly by vibrational bands originating from X-H vibrations that are highly localized [59] and therefore, do not depend much on the chain vibrations of the polymer. Hence, the length of the polymer chain should have much lesser meaning for the NIR spectra. Noteworthy, such assumption would not be valid for IR spectra, particularly for fingerprint region and low-lying vibrational modes, where chain deformations are essential contributions to the vibrational spectrum of polymers [60]. Accordingly, to bring the complexity of the vibrational problem to a level suitable for anharmonic calculations, in this work the models representing fragments of polymer counting from 1 to 6 units were used (Fig. 1). The exact size of each of the models was selected in order to maintain the total number of the electrons below ca. 140 electrons (Table S1 in Supplementary Material). Accordingly, POM is represented by 6 units, PLA and PVC each by 3 units, PMMA and PS each by 2 units, while the rest of the models count 1 unit (Table S1). Note, in the case of the copolymers, ABS and EVAC, the simplest models were used in which just one of each of the monomer species were included. As it will be demonstrated, such approach did not reduce the agreement with the experimental spectra as compared with the other seven studied polymers.

The models defined in such way required attachment of the terminal moieties in order to obtain closed shell systems. However, the polymers with the units bearing oxygen atoms in the main chain, i.e. POM, PLA, PET and PC, required further considerations. Because of the aim to approximate NIR spectra, the most straightforward termination by the addition of hydrogen atoms to the terminal bonds was not used in this case. High anharmonicity of O-H vibrations would introduce artificial intense peaks to the models NIR spectra strongly reducing their agreement with the experiment. Therefore, we used CH_3_ groups to terminate the models. Additionally, special attention was given to validate whether this approach resulted in artificial enhancement of the bands originating from CH_3_ groups in the simulated NIR spectra. The models used for this study, after geometry optimization, are presented in Supplementary Material (Tables S2-S10).

### 3.2 Theoretical NIR spectra of organic polymers

**Fig. 2:**
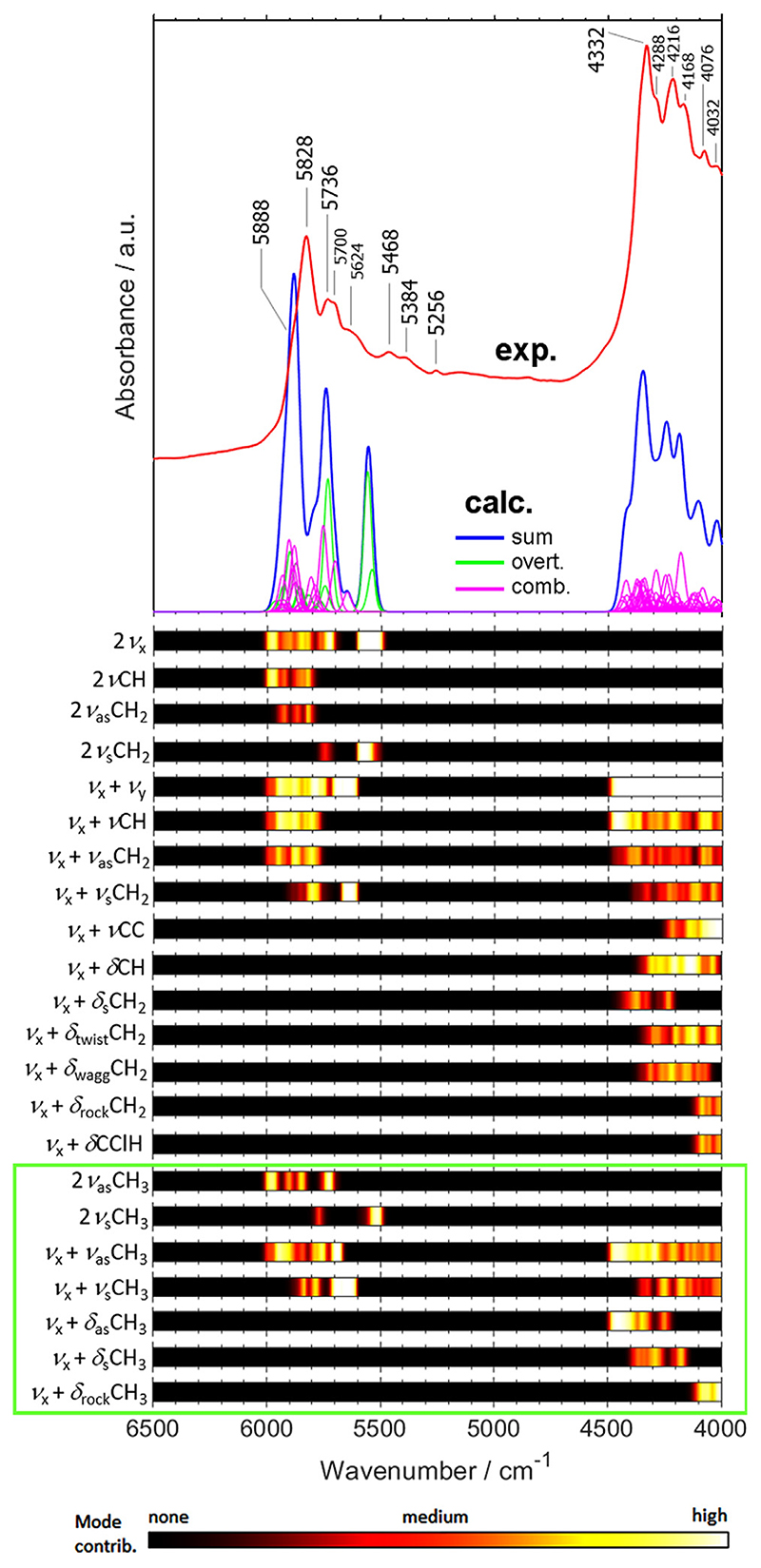
Experimental and simulated (B3LYP/6-311+G(df,pd)) NIR spectrum of PVC.

Firstly, one should give attention to the NIR spectrum of PVC (Fig. 2). This polymer has relatively simple structure, and importantly, the low-frequency vibrations of C-Cl moiety (resulting from a very high reduced mass of this oscillator) should not be expected to be meaningful in NIR spectrum. Hence, one would expect the NIR spectrum of PVC to be relatively less abundant in features compared with the rest of the polymers investigated in this work. Accordingly, the NIR spectrum of PVC exhibits two clearly distinct regions of absorption, in 6000–5250 cm^−1^ and in 4500–4000 cm^−1^ (Fig. 2). The theoretical spectrum unveils the origins of the absorption bands in high detail. In both respective regions, the major contribution to the observed lineshape stems from numerous combination bands, while the overtones only show meaningful presence in the former one. A note on the theoretical intensity of the overtone bands at ca. 5600–5500 cm^−1^ will be given separately. This dominance of the combination bands to the total intensity of NIR spectra of polymers can be presented by calculating the percentage of the integral intensity contributed to the theoretical spectrum by the overtones and combination bands (Table S11). Only for PC the contribution from overtones reaches nearly 25% of the integral intensity, resulting from relatively strong overtone bands of CH stretching modes in the aromatic rings (Table S11).

**Fig. 3:**
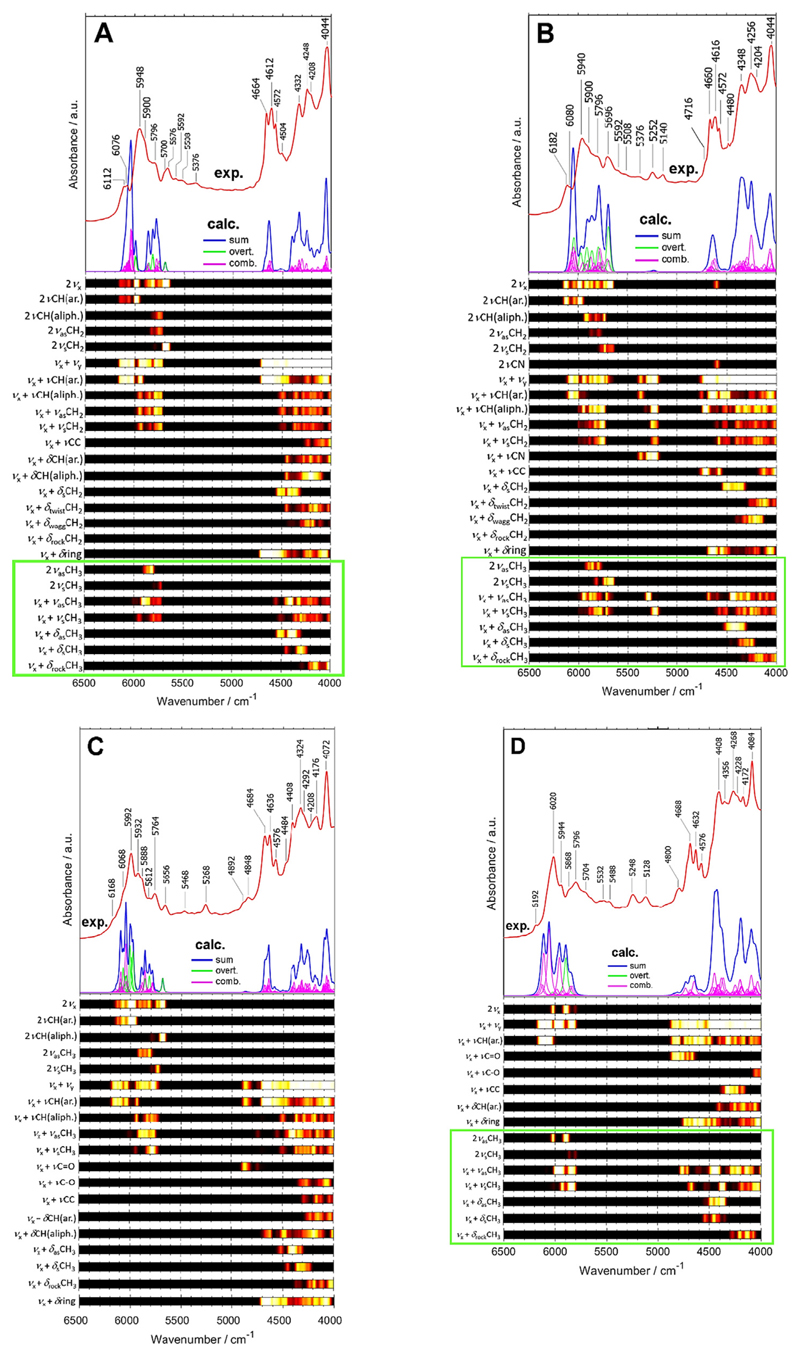
Experimental and simulated (B3LYP/6-311+G(df,pd)) NIR spectrum of PS (A), ABS (B), PC (C) and PET (D).

**Fig. 4:**
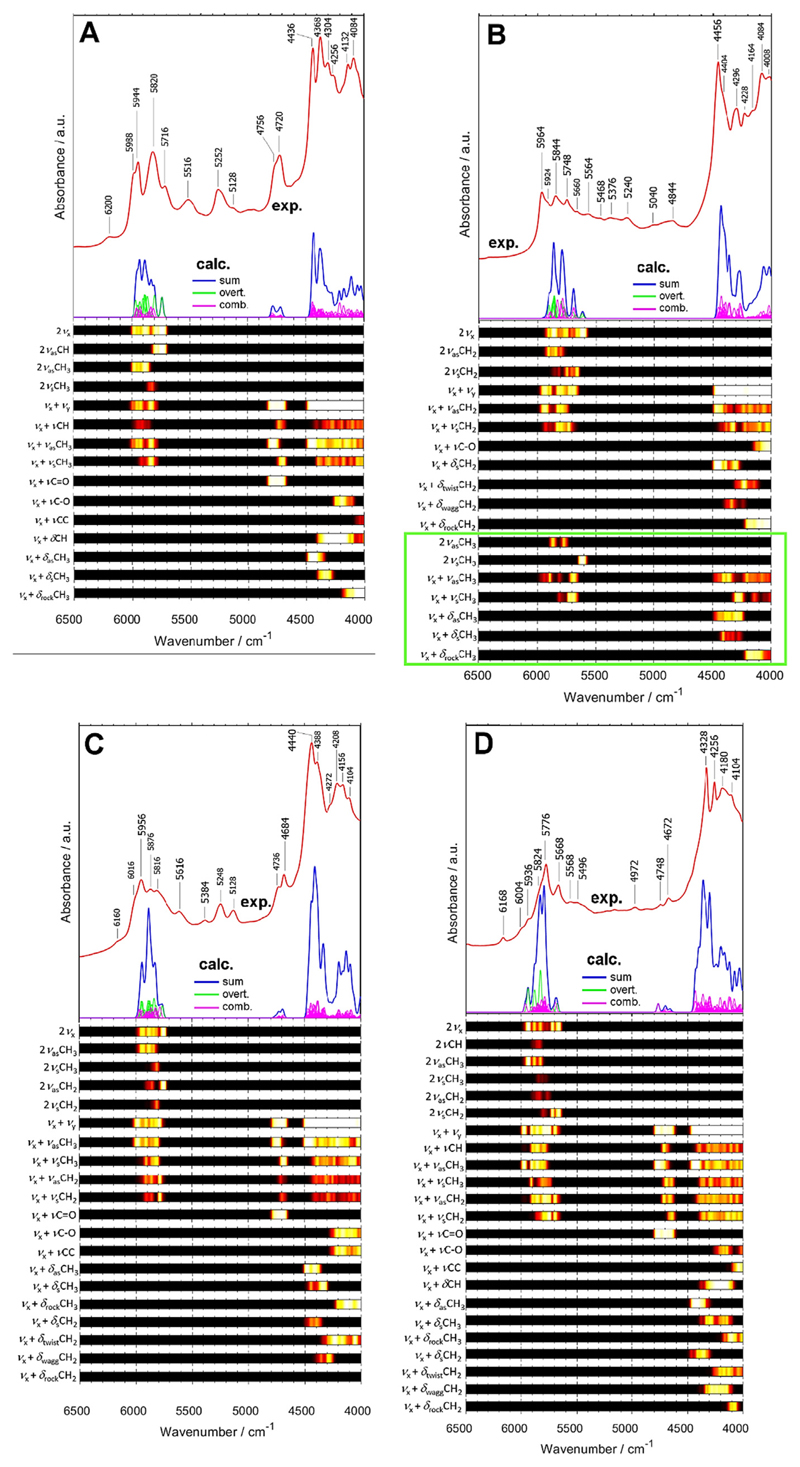
Experimental and simulated (B3LYP/6-311+G(df,pd)) NIR spectrum of PLA (A), POM (B), PMMA (C) and EVAC (D).

Strong band overlapping, an intrinsic feature of NIR spectra, can easily be noted throughout the entire investigated spectral region; this is particularly evident in ca. 4500–4000 cm^−1^. For a better view of the details, the overlapping of NIR bands of PVC is presented in enlarged Fig. S1 (Supplementary Material). As expected, the contribution from C-Cl moiety to NIR spectrum of PVC is relatively insignificant, with only moderate influence in the narrow low-wavenumber fragment of the NIR spectrum, i.e. at ca. 4100–4000 cm^−1^ (Fig. 2). Note, the 5000 to 4000 cm^−1^ region may serve as a particularly sensitive benchmark for the accuracy of the theoretical spectrum. The degree of band overlapping observed there is extensive (Fig. 2 and Fig. S1); thus, even minor inaccuracy in the calculated positions and intensities of individual peaks would result in visible distortion of the summarized theoretical lineshape from the experimental one. Notwithstanding, the agreement between the simulated and experimental lines is remarkably good for all PVC as well as for the remaining 8 investigated polymers (Figs. 3 and 4), which validates the suitability of the calculated spectra.

In addition to the band overlapping, another feature of NIR spectrum distinct from the better researched MIR absorption can be confirmed in the case of PVC. In addition, the specific vibrational contributions tend to be dispersed over the wavenumber axis rather than appearing in relatively narrow, well-defined wavenumber regions as it occurs in IR spectra. Hence, neither the tabularized band assignments as commonly accepted for IR spectroscopy, nor the group frequencies concept are applicable to NIR spectra. For these reasons, the assignments that reflect the nature of NIR spectra in a proper way may be represented in the form of an intensity map of the vibrational contributions presented for the selected meaningful transitions (Fig. 2) [39].

In the presented calculations, the computational complexity prevented the treatment of three quanta transitions, i.e. the second overtones and ternary combinations. Importantly, it should be noted that the approximation of the NIR spectra by two quanta transitions (i.e. first overtones, binary combinations) was demonstrated to be sufficient for several smaller molecules, for which the agreement between the theoretical spectrum obtained this way and the experimental one was very good [30,44]. The third quanta bands, in particular the ternary combinations, are numerous but universally weak. Therefore, the NIR absorption stemming from those transitions is rather featureless, mostly manifested by weak and broad features rarely protruding from the NIR baseline [30,44]. Hence, their importance for the interpretation for NIR spectra is low. One of the possible exceptions may be formed by the C=O stretching vibration, for which the second overtone may be identified in the NIR spectra of some compounds including polymers [53,61]. Attention will be given to this problem in the case of polymer bearing C=O functionality (i.e. EVAC, PC, PET, PLA, PMMA). In the present case, the relative simplicity of the NIR spectrum of PVC enables to confirm that a similar regularity occurs here. A very good agreement, with the exception of ca. 5800–5500 cm^−1^ region (explained beneath), can be concluded between the experimental and the theoretical NIR spectrum of PVC consisting of the first overtones and binary combination bands (Fig. 2). Therefore, the discussed approach yields full capacity to provide reliable theoretical NIR spectra for detailed assignments in the case of polymers as well.

Since no CH_3_ groups are present in side chains of PVC, it is the ideal case for the evaluation of the effect that the artificial augmentation in CH_3_ groups may impose on the calculated spectrum. The contributions from CH_3_ groups are highlighted with a green frame in Figs. 2–4 for the polymers where such augmentation occurs. In spite of the augmentation, the agreement between the theoretical NIR spectrum of PVC model designed this way and the experimental spectrum is good with clearly identifiable overestimation of the theoretical intensity at ca. 5600–5500 cm^−1^, and to lesser extent, in the region of 5700–5600 cm^−1^ (Fig. 2). Hence, this effect does not impair the interpretability of the spectra. In the case of all the remaining polymers for which the terminal CH_3_ groups co-exist with the side chain CH_3_ groups (i.e. EVAC, PC, PLA, PMMA), the extent of this effect on intensity should be even lesser. It should be highlighted that it is not only the strong overtones of CH_3_ stretching modes but also of the stretching vibrations of CH_2_ groups that contribute to this elevated theoretical band of PVC at ca. 5600–5500 cm^−1^ (Fig. 2). The overestimation of the intensities of the CH_3_ and CH_2_ overtones is known to be present in VPT2 calculations for other systems [41,62]. Therefore, the overestimated spectral intensity at ca. 5700–5500 cm^−1^ observed in the calculated spectra can reliably be taken account when interpreting the NIR spectral lineshape of the polymers analyzed here.

The exhaustive NIR bands assignments for the nine polymers (ABS, EVAC, PC, PET, PLA, PMMA, POM, PS, PVC) are presented in Figs. 2–4. Based on these results, more generalized spectra-structure correlations for polymers can be derived. At glance, one should note that the upper NIR region of polymers (ca. 6500–5500 cm^−1^) is mostly populated by combination bands originating from νCH, νCH_2_, νCH_3_ and overtones of νCH, νCH_2_, νCH_3_. The presence of aromatic rings in the structure of polymer draws keen interest to elucidate the NIR spectral footprint of this structure (Fig. 3). Firstly, a very sharp separation between the NIR bands that originate from the stretching modes of C-H aliphatic and aromatic functionalities. Interestingly, this can be observed both for the overtones and the combination bands (Fig. 3). It is reasonable, given the well-known difference between the position of the fundamental νC-H bands for aromatic and aliphatic moieties, respectively. This difference is enhanced in NIR spectra. In addition, this difference is also manifested in the positions of the respective combination bands in the lower wavenumber fragment of NIR spectra (ca. 4700–4000 cm^−1^).

Next, as consistently observed in Fig. 3 for the polymers that feature aromatic rings (ABS, PC, PET and PS) a characteristic feature of relatively strong intensity appears between 4700 and 4500 cm^−1^ with three peak maxima albeit positioned at different wavenumbers for those four polymers. That feature originates from the combination bands involving ring deformation modes and several other modes, mostly CH stretching modes. The easily recognizable structure, strong intensity and the separation of this feature from other NIR absorption regions of polymers make it highly characteristic marker of the presence of an aromatic ring in the polymer structure (Fig. 3). A relatively consistent shape of this structure for all four different polymers suggest that the ring deformation vibrations tends to quite selectively couple with νC-H (in ring) while disregarding other modes, thus not being sensitive to the chemical neighborhood. Furthermore, the presence of an aromatic ring in the structure of a polymer leads to the formation of a sharp peak at ca. 4100–4000 cm^−1^ (combination bands involving δring mode). It is the most intense absorption feature in the NIR region for those polymers, and therefore, also makes it very useful for the analysis of these compounds.

The manifestation of C=O functionality in NIR spectrum is highly interesting, as it is known that for certain molecules the stretching second overtone can be identified as well-resolved peak (e.g. ref. [53]). One can identify characteristic single peak or two partially overlapping peaks (at ca. 5250–5120 cm^−1^1 in NIR spectra of the polymers bearing C=O functionality (EVAC, PC, PET, PLA, PMMA). This absorption was not reproduced in the calculated NIR spectra. Given this fact it is concluded that this feature is contributed by the second overtone of C=O stretching mode, which remains in agreement with the literature [53,61]. For the polymers that manifest two peaks in this region (EVAC, PET, PMMA), it may be suggested that the structural differences in the polymer sample resulting in the C=O moieties located in different neighborhoods could be responsible for the observed band splitting. However, as it will be shown for ABS, the combination bands involving νC≡N mode appear in the same region and result in a spectral line of similar shape. Therefore, the specificity of this region is lowered if presence of both kinds of polymers is suspected in the sample.

Further valuable conclusions can be drawn for the combination bands involving C=O modes. Interestingly, the NIR footprint of C=O moiety is easily identifiable in NIR spectra but decisively less consistent compared with that of the aromatic ring. The combination bands involving νC=O mode are located in the region free from other meaningful bands, i.e. in ca. 4900–4600 cm^−1^. Noteworthy, this holds for the polymer where C=O moieties and aromatic rings co-exist (i.e. PET and PC). For these two cases, the intensity of the νC=O combinations bands is relatively enhanced and blue-shifted compared with the polymers bearing C=O groups but lacking an aromatic ring (i.e. EVAC, PLA and PMMA; Fig. 4). The features arising from the combinations of νC=O and other modes are intense enough to be easily identified; however, their position and shape differ widely among the studied polymers. Hence, the νC=O mode does not tend to couple selectively with any particular mode of the moieties in the nearest vicinity. The effects of the coupling with stretching vibrations of C-H, CH_2_ and CH_3_ moieties in proximity can be observed. Consequently, this less local character of the νC=O combinations leads to the increased sensitivity of these NIR bands to the chemical neighborhood. Thus, the νC=O bands in NIR spectra may be more sensitive towards the structural changes in the polymer.

Noteworthy, νC≡N fundamental band is highly characteristic in IR spectra, where it appears as a strong and sharp peak at ca. 2250 cm^−1^ [36]. As this peak is located in the IR region typically free from any other meaningful fundamental bands, it is useful as a structural marker in polymers as well [63]. One could expect that a similar manifestation of C≡N group present in a polymer will be seen in its NIR spectrum. However, the NIR spectrum of ABS demonstrates that this is not the case (Fig. 3B). In NIR spectrum of ABS in approximate position where the 2νC≡N band would be expected to be found, the strong abortion feature appears that originates from combination bands involving ring deformation modes as discussed above. The calculated spectrum unveils that the 2νC≡N band contributing into this feature is very weak (constitutes less than 0.5% to the integral intensity in 5000–4000 cm^−1^ of the theoretical spectrum of ABS; Table S11), which would make it less useful even if the absence of strong absorption due to the aromatic ring. Thus, in the NIR spectrum the νC≡N mode forfeits its specificity known in IR spectra.

POM exhibits a number of weak overlapping bands resulting in a continuous absorption between ca. 5800–4700 cm which were not reproduced in the theoretical NIR spectra (Fig. 4). As a viable reason, it could be suggested that these bands originate from three quanta transitions, which could not be included in the calculations presented in this work. It is also possible that the high conformational flexibility of POM results in strongly varying local conditions for the CH_2_ vibrations, causing red-shift of some of the bands from their predicted positions at ca. 5900–5600 cm^−1^. These could at least partially contribute to the observed broad absorption feature of POM.

Finally, it should be noted, that there is no clear correspondence between the number of units included in the model of polymer and the resulting accuracy of the calculated spectrum among the 9 systems investigated here. Thus, this confirms the assumption based on the previous studies, that the effect of the chemical neighborhood, here extended to the length of the polymer chain, is relatively less significant as the factor shaping NIR spectra, which stands in contrast to the regularities established for IR spectra [44,45].

## 4 Conclusions

The problem of the complex nature of NIR spectra and the difficulties in their direct interpretation can be successfully addressed with aid of quantum chemical calculations. Polymers form additional complication because of the molecular size, but also the uncertainty of their structure, i.e. chain conformation and polymorphism. However, the character of the majority of the NIR bands of polymers makes them less sensitive to the distant chemical neighborhood. For this reason, accurate simulation of NIR spectra of polymers, fully suitable for band identifications, can be achieved with the use of simplified models counting few (1–6) units of the polymer; this ways, anharmonic calculations become feasible for such systems. Successful assignments for 9 different polymers leading to insightful generalizations of the spectra-structure correlations were presented here.

The same mechanism makes NIR spectra highly useful for structural identification of polymers, even more so than it is accepted for IR spectra. With aid of accurate quantum chemical calculations of overtones and combination bands, NIR spectroscopy can demonstrate superiority in chemical specificity and structural selectivity. The effects related to the longer-range chemical neighborhood are not pronounced, instead the presence of the functional groups is clearly manifested in NIR spectra. This can be most prominently identified for the aromatic rings, for which the coupling between the CH stretching and ring deformation modes gives rise to highly characteristic NIR bands. The combination transitions localized on the aromatic ring are relatively independent from the remaining molecular fragments. Despite distinct differences in their structure, ABS, PC, PET and PS polymers all manifest similar, well-resolved and intense absorption feature at ca. 4500–4700 cm^−1^ in the region free from other meaningful bands. In addition, the presence of an aromatic ring can be unveiled in these spectra at ca. 4080–4040 cm^−1^; it is manifested as a sharp and intense peak, albeit arising from a broad absorption structure.

A number of other essential correlation between the NIR spectra and structure of polymers were identified. The presence of νC=O functionality gives rise to intense combination bands located in the region free from other meaningful absorption (ca. 4900–4600 cm^−1^) even if aromatic rings are present in the structure of the polymer (i.e. PET and PC). However, the νC=O mode coupled with the vibrations of the moieties in proximity. Thus, the position and shape of the combination bands depends relatively more on the chemical neighborhood that it was concluded for aromatic ring. The contribution from C-Cl moiety to NIR spectrum is relatively insignificant, with only moderate influence in the narrow low-wavenumber fragment of the NIR spectrum, i.e. at ca. 4100–4000 cm^−1^. Thus, the resulting spectrum of PVC is relatively less rich in features than the other polymers examined here. Possible pitfalls from attempting to identify NIR bands based on the correlations of peak positions and intensities observed in better explained IR spectra should be noted. In the present case, such occurrence as noted for 2νC≡N band of ABS. In the region where this band could be expected to appear based on its fundamental, NIR absorption originates from the combination bands of ring deformation with other modes.

## Supplementary Material

Supplementary data to this article can be found online at https://doi.org/10.1016/j.saa.2021.120085.

SI

## Highlights

- NIR spectra simulation by anharmonic VPT2 calculations.
- Near-infrared (NIR) “fingerprint” of nine organic polymers.
- ABS, EVAC, PC, PET, PLA, PMMA, POM, PS, and PVC polymers studied.
- Interpretation of NIR spectra.
- Spectra-structure correlations and footprint of structural motifs.
