# Supplementary material for: Spectra-structure correlations in NIR region of polymers from quantum chemical calculations. The cases of aromatic ring, C=O, C≡N and C-Cl functionalities
Source: Spectrochim Acta A Mol Biomol Spectrosc. Author manuscript; Available in PMC 2024 Dec 5. (PMC7616891; doi:10.1016/j.saa.2021.120085)
Supplement: SI [file EMS137681-supplement-SI.docx]

**Spectra-structure correlations in NIR region of polymers from quantum chemical calculations. The cases of aromatic ring, C=O, C≡N and C-Cl functionalities**

**Supplementary Material**

Krzysztof B. Beć^1*^, Justyna Grabska^1^, Jovan Badzoka^1^, Christian W. Huck^1^

*^1^ Institute of Analytical Chemistry and Radiochemistry, University of Innsbruck, Innrain 80-82, 6020 Innsbruck, Austria*

***Corresponding author**

Dr. Krzysztof B. Beć

Email: Krzysztof.Bec@uibk.ac.at

**
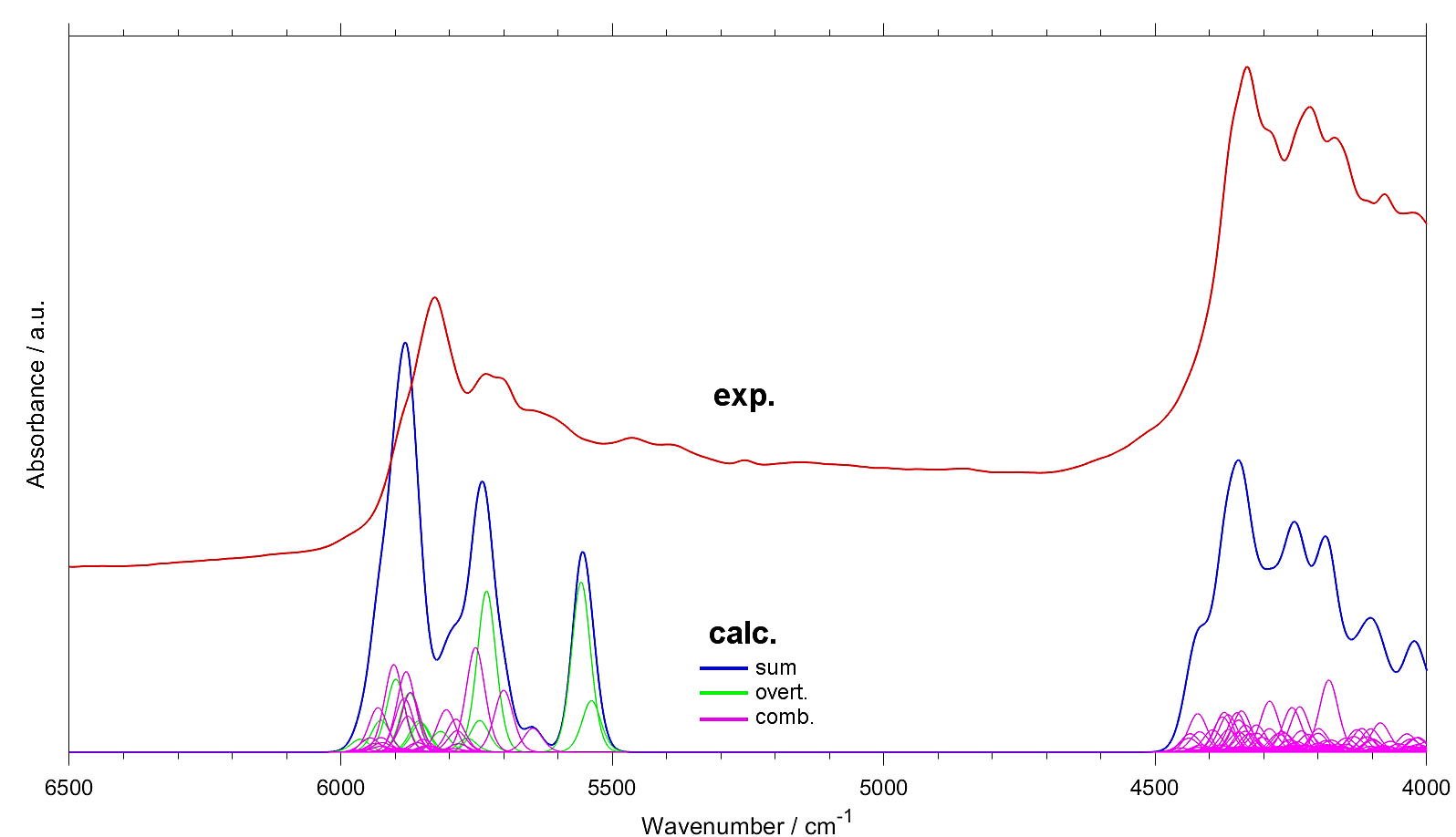
**

**Figure S1. Experimental and theoretical NIR spectrum of PVC.**

**Table S1. The parameters of the models used to represent polymer structures.**

| polymer | units | atoms | electrons |
| --- | --- | --- | --- |
| ABS | 1 | 35 | 116 |
| EVAC | 1 | 32 | 96 |
| PC | 1 | 35 | 136 |
| PET | 1 | 24 | 102 |
| PLA | 3 | 28 | 108 |
| PMMA | 2 | 38 | 126 |
| POM | 6 | 29 | 106 |
| PS | 2 | 40 | 130 |
| PVC | 3 | 22 | 105 |

**Table S2. Optimized structure of the model representing ABS in Cartesian coordinates.**

C 0.054663000 -0.719978000 -0.289023000

H 0.140741000 -1.166742000 0.705028000

C -0.005152000 0.782768000 -0.108516000

C -0.032257000 1.340311000 1.168591000

C -0.054690000 1.640574000 -1.209718000

C -0.105947000 2.718592000 1.345610000

H -0.001395000 0.686366000 2.031447000

C -0.127557000 3.017458000 -1.038525000

H -0.031752000 1.232276000 -2.213146000

C -0.153587000 3.562154000 0.242182000

H -0.126726000 3.131327000 2.346467000

H -0.162392000 3.666862000 -1.904536000

H -0.210041000 4.634853000 0.376836000

C -1.229357000 -1.258875000 -0.937898000

H -1.164098000 -2.347495000 -1.017616000

H -1.321277000 -0.868143000 -1.955075000

C -2.521406000 -0.888989000 -0.185538000

H -2.586573000 0.201975000 -0.134100000

C -3.773705000 -1.431101000 -0.890237000

H -4.680360000 -1.146388000 -0.356643000

H -3.825590000 -1.026439000 -1.901866000

H -3.740999000 -2.519568000 -0.954365000

C -2.462878000 -1.364642000 1.197622000

N -2.405646000 -1.752360000 2.280895000

C 1.292291000 -1.168293000 -1.095734000

H 1.268352000 -2.256709000 -1.197991000

H 1.221047000 -0.754682000 -2.108383000

C 2.584814000 -0.748178000 -0.465971000

H 2.749170000 0.323034000 -0.378074000

C 3.506441000 -1.583845000 0.001925000

H 3.331565000 -2.655056000 -0.082341000

C 4.791765000 -1.173086000 0.650160000

H 4.861499000 -1.565900000 1.668999000

H 5.653805000 -1.565420000 0.102111000

H 4.883929000 -0.086849000 0.697406000

**Table S3. Optimized structure of the model representing EVAC in Cartesian coordinates.**

C -4.687069000 -0.353893000 0.151753000

H -5.598638000 -0.886293000 -0.127070000

H -4.700733000 0.616793000 -0.350480000

H -4.724402000 -0.168568000 1.228165000

C -3.437619000 -1.147628000 -0.224606000

H -3.438825000 -1.344537000 -1.302100000

H -3.464688000 -2.127293000 0.264622000

C -2.141831000 -0.431397000 0.153415000

H -2.119004000 0.548072000 -0.333742000

H -2.132388000 -0.238294000 1.231460000

C -0.890852000 -1.218275000 -0.231214000

H -0.879484000 -1.398269000 -1.311375000

H -0.914529000 -2.200546000 0.251672000

C 0.411372000 -0.539545000 0.167442000

H 0.400097000 -0.300401000 1.231874000

C 1.650112000 -1.354015000 -0.174480000

H 1.671543000 -1.529124000 -1.255436000

H 1.545398000 -2.334637000 0.301191000

C 2.960192000 -0.708645000 0.274541000

H 2.919901000 -0.523929000 1.352390000

H 3.061402000 0.270097000 -0.201677000

O 0.495865000 0.720336000 -0.558944000

C 0.527826000 1.874401000 0.135097000

C 0.609075000 3.050315000 -0.802181000

H -0.260328000 3.057534000 -1.461245000

H 0.648485000 3.972154000 -0.228618000

H 1.495337000 2.962337000 -1.432012000

O 0.494658000 1.949110000 1.338284000

C 4.179684000 -1.565492000 -0.058767000

H 5.104263000 -1.085200000 0.267115000

H 4.121403000 -2.541544000 0.429879000

H 4.255664000 -1.738892000 -1.135328000

**Table S4. Optimized structure of the model representing PC in Cartesian coordinates.**

C 3.770973000 -0.162717000 -0.120467000

C 2.951741000 0.785149000 0.498737000

C 3.205299000 -1.384698000 -0.477464000

C 1.612866000 0.527097000 0.754073000

H 3.361408000 1.744224000 0.789452000

C 1.863462000 -1.659468000 -0.233212000

H 3.820834000 -2.136716000 -0.956394000

C 1.081820000 -0.697268000 0.378308000

H 0.986124000 1.268666000 1.228852000

H 1.423334000 -2.608452000 -0.508379000

C -1.227086000 -0.284244000 0.154888000

C -3.588216000 -0.226815000 0.220610000

C -3.857842000 1.107445000 0.485417000

C -4.533662000 -1.058371000 -0.357104000

C -5.106980000 1.616102000 0.150319000

H -3.102904000 1.737203000 0.932920000

C -5.781505000 -0.538894000 -0.683738000

H -4.286235000 -2.095199000 -0.540040000

C -6.069753000 0.798128000 -0.433035000

H -5.326904000 2.657258000 0.348069000

H -6.526266000 -1.181604000 -1.135026000

H -7.040445000 1.201749000 -0.689825000

O -2.381059000 -0.817746000 0.600273000

O -0.242017000 -1.036997000 0.676734000

O -1.104064000 0.663681000 -0.564737000

C 5.402905000 1.304357000 -1.364365000

H 5.023477000 2.229706000 -0.925025000

H 6.457865000 1.456512000 -1.603104000

H 4.861005000 1.130380000 -2.295293000

C 6.019642000 0.355645000 0.899857000

H 5.917960000 -0.493631000 1.577467000

H 7.080931000 0.499495000 0.685283000

H 5.661731000 1.245513000 1.422740000

C 5.232783000 0.124879000 -0.397362000

H 5.646509000 -0.764577000 -0.882637000

**Table S5. Optimized structure of the model representing PET in Cartesian coordinates.**

C 4.987918000 0.578113000 -0.000072000

H 5.312975000 0.035696000 0.887370000

H 5.386986000 1.588549000 -0.000011000

O 3.560510000 0.728080000 -0.000071000

C 2.850998000 -0.415957000 0.000023000

O 3.362102000 -1.510794000 0.000106000

C 1.380995000 -0.173653000 0.000017000

C 0.839107000 1.113514000 0.000043000

C 0.535753000 -1.284069000 -0.000008000

C -0.535744000 1.284046000 0.000041000

H 1.497095000 1.969987000 0.000062000

C -0.839099000 -1.113537000 -0.000015000

H 0.974527000 -2.272234000 -0.000021000

C -1.380987000 0.173629000 0.000009000

H -0.974517000 2.272212000 0.000064000

H -1.497085000 -1.970012000 -0.000038000

C -2.850990000 0.415940000 0.000004000

O -3.362102000 1.510773000 0.000039000

O -3.560530000 -0.728089000 -0.000057000

H 5.312976000 0.035805000 -0.887564000

C -4.987925000 -0.578030000 -0.000030000

H -5.312937000 -0.035181000 -0.887229000

H -5.387025000 -1.588451000 -0.000563000

H -5.312985000 -0.036111000 0.887722000

**Table S6. Optimized structure of the model representing PLA in Cartesian coordinates.**

C 3.059671000 -0.560904000 0.105634000

O 2.586717000 -1.532721000 -0.427045000

O 2.521662000 0.676018000 -0.005716000

C 1.291934000 0.763175000 -0.732032000

H 1.406743000 0.282432000 -1.703823000

C 0.951393000 2.238040000 -0.878684000

H 1.736699000 2.742026000 -1.441779000

H 0.002815000 2.348373000 -1.402157000

H 0.861644000 2.702614000 0.102929000

C 0.189908000 0.040017000 0.029853000

O -0.801279000 -0.299272000 -0.816197000

O 0.176570000 -0.166110000 1.212899000

C -1.991087000 -0.813437000 -0.215370000

H -1.716070000 -1.545424000 0.547782000

C -2.807510000 -1.467573000 -1.321560000

H -2.212340000 -2.241043000 -1.805678000

H -3.706743000 -1.929180000 -0.913695000

H -3.097459000 -0.729229000 -2.070206000

C -2.764845000 0.322307000 0.467306000

O -2.444628000 1.475808000 0.321816000

C -3.959963000 -0.094585000 1.290886000

H -4.815823000 -0.252899000 0.628806000

H -3.776103000 -1.027807000 1.825091000

H -4.209178000 0.698527000 1.992566000

C 4.281522000 -0.540873000 0.977483000

H 4.823746000 -1.476070000 0.867352000

H 4.919611000 0.306665000 0.729713000

H 3.963983000 -0.425787000 2.015958000

**Table S7. Optimized structure of the model representing PMMA in Cartesian coordinates.**

C 1.211976000 -0.820413000 0.646511000

C 0.003649000 -0.025598000 1.195860000

H 0.396529000 0.712491000 1.897208000

H -0.608614000 -0.720497000 1.771598000

C -0.930789000 0.727979000 0.216177000

C -1.904560000 1.560903000 1.100375000

H -1.298573000 2.311348000 1.615278000

H -2.314054000 0.902374000 1.869257000

C 0.824900000 -1.808566000 -0.458165000

H 1.674613000 -2.432461000 -0.733055000

H 0.027489000 -2.459156000 -0.095041000

H 0.478849000 -1.310290000 -1.361323000

C 2.307012000 0.158376000 0.218598000

O 2.666407000 1.102035000 0.880685000

O 2.878259000 -0.164027000 -0.955899000

C 3.936430000 0.700951000 -1.395293000

H 4.276989000 0.288359000 -2.340946000

H 3.563239000 1.715879000 -1.530992000

H 4.745726000 0.715125000 -0.665843000

C -0.162441000 1.648837000 -0.735215000

H 0.445393000 2.347287000 -0.160447000

H 0.489850000 1.084865000 -1.400687000

H -0.844434000 2.209607000 -1.370969000

C -1.784134000 -0.235208000 -0.605481000

O -2.411164000 -1.142254000 0.174281000

O -1.940208000 -0.192392000 -1.800611000

C -3.264439000 -2.070674000 -0.508165000

H -3.682289000 -2.708905000 0.265781000

H -4.056115000 -1.543712000 -1.040319000

H -2.690330000 -2.660038000 -1.223328000

C -3.052333000 2.246055000 0.361890000

H -2.694062000 2.944269000 -0.395270000

H -3.702177000 1.522945000 -0.135360000

H -3.666682000 2.809754000 1.066232000

C 1.818414000 -1.606152000 1.831827000

H 2.718833000 -2.141697000 1.524143000

H 2.077438000 -0.934940000 2.650576000

H 1.093908000 -2.338450000 2.192349000

**Table S8. Optimized structure of the model representing POM in Cartesian coordinates.**

C -3.906158000 1.906734000 -0.316450000

H -3.713657000 2.871803000 0.148408000

H -4.947291000 1.873335000 -0.660770000

H -3.245820000 1.778951000 -1.179034000

O -3.667349000 0.917280000 0.672770000

C -3.928151000 -0.381688000 0.235644000

H -3.897406000 -1.016385000 1.123835000

H -4.911713000 -0.432247000 -0.246304000

O -3.001056000 -0.832790000 -0.733624000

C -1.923834000 -1.591993000 -0.238811000

H -1.345523000 -1.878990000 -1.118814000

H -2.285097000 -2.476513000 0.293583000

O -1.129194000 -0.907423000 0.698385000

C -0.411441000 0.189230000 0.179343000

H -1.055177000 0.830492000 -0.420670000

H -0.027584000 0.725529000 1.047073000

O 0.634896000 -0.217253000 -0.676092000

C 1.733425000 -0.799886000 -0.009458000

H 2.335584000 -1.271686000 -0.787093000

H 1.401079000 -1.531993000 0.727756000

O 2.485730000 0.152803000 0.706807000

C 3.262698000 1.007914000 -0.111502000

H 3.574819000 1.824059000 0.548590000

H 2.670761000 1.387077000 -0.946106000

O 4.364737000 0.363880000 -0.677454000

C 5.353980000 -0.019714000 0.267561000

H 4.965866000 -0.754186000 0.978022000

H 6.175968000 -0.457167000 -0.295774000

H 5.719973000 0.852358000 0.823001000

**Table S9. Optimized structure of PS in Cartesian coordinates.**

C 0.916309000 0.603639000 1.491710000

H 0.244147000 -0.185157000 1.839642000

C 0.053462000 1.681760000 0.815908000

H 0.688676000 2.522924000 0.520662000

H -0.648762000 2.076787000 1.556920000

C -0.722327000 1.201244000 -0.420650000

H 0.011313000 0.880130000 -1.166327000

C -1.530109000 2.356431000 -1.037824000

H -0.851537000 3.199418000 -1.202218000

H -2.276642000 2.699248000 -0.314057000

C 1.890150000 -0.037416000 0.524555000

C 2.837891000 0.725397000 -0.161510000

C 1.856594000 -1.411480000 0.292968000

C 3.721616000 0.133007000 -1.055143000

H 2.885392000 1.795696000 -0.000312000

C 2.739938000 -2.011343000 -0.599034000

H 1.119744000 -2.014883000 0.808863000

C 3.675508000 -1.240138000 -1.278571000

H 4.447722000 0.742883000 -1.578605000

H 2.694371000 -3.080801000 -0.764487000

H 4.363081000 -1.702765000 -1.975329000

C -1.597270000 0.004429000 -0.111814000

C -1.419312000 -1.200069000 -0.789825000

C -2.601581000 0.075587000 0.856082000

C -2.216425000 -2.305833000 -0.511369000

H -0.639581000 -1.272754000 -1.537801000

C -3.400618000 -1.025025000 1.140041000

H -2.762163000 1.000728000 1.396551000

C -3.210195000 -2.222782000 0.456827000

H -2.059563000 -3.232002000 -1.050605000

H -4.173290000 -0.949124000 1.895290000

H -3.831875000 -3.081333000 0.677395000

C -2.216893000 1.987300000 -2.350146000

H -2.933025000 1.176546000 -2.206468000

H -2.753574000 2.841616000 -2.767287000

H -1.486363000 1.656023000 -3.092742000

C 1.639100000 1.177522000 2.717248000

H 0.920891000 1.586977000 3.431506000

H 2.223312000 0.406488000 3.222120000

H 2.322702000 1.979975000 2.430576000

**Table S10. Optimized structure of the model representing PVC in Cartesian coordinates.**

Cl -3.678688000 -0.789311000 -0.229753000

C -1.191936000 0.065955000 -1.087938000

H -1.207168000 -0.963909000 -1.444468000

H -0.927280000 0.703073000 -1.943534000

C -2.537018000 0.468528000 -0.593065000

C -0.054172000 0.235658000 -0.072228000

H -0.044675000 1.258000000 0.300385000

Cl -0.395415000 -0.775637000 1.412340000

C 1.294977000 -0.136177000 -0.671026000

H 1.252735000 -1.162159000 -1.040682000

H 1.458474000 0.514351000 -1.536054000

C 2.479199000 0.008519000 0.277617000

H 2.414641000 -0.729372000 1.073020000

Cl 3.988340000 -0.462070000 -0.650967000

C 2.661016000 1.398823000 0.853467000

H 2.718347000 2.146742000 0.060507000

H 1.821184000 1.641019000 1.508420000

H 3.572725000 1.454324000 1.446419000

C -2.811833000 1.808711000 -0.018185000

H -3.855421000 2.096665000 -0.156196000

H -2.606602000 1.839916000 1.061226000

H -2.180382000 2.560544000 -0.498418000

**Table S11. The relative contribution (in %) of the first overtones (2ν) and binary combination bands (ν + ν) to the integral intensity of the simulated NIR spectrum of polymers.**

|  | 6500 – 4000 cm^-1^ | | 5000 – 4000 cm^-1^ | | 6500 – 5000 cm^-1^ | |
| --- | --- | --- | --- | --- | --- | --- |
| Polymer | 2ν | ν+ν | 2ν | ν+ν | 2ν | ν+ν |
| ABS | 19,85 | 80,15 | 0,46 | 99,54 | 44,04 | 55,97 |
| EVAC | 9,33 | 90,67 | 0,00 | 100,00 | 28,72 | 71,28 |
| PC | 24,91 | 75,09 | 0,00 | 100,00 | 55,21 | 44,79 |
| PET | 5,11 | 94,89 | 0,00 | 100,00 | 15,67 | 84,33 |
| PLA | 21,37 | 78,63 | 0,00 | 100,00 | 63,49 | 36,51 |
| PMMA | 14,66 | 85,34 | 0,00 | 100,00 | 50,80 | 49,20 |
| POM | 17,11 | 82,89 | 0,00 | 100,00 | 56,05 | 43,95 |
| PS | 11,81 | 88,19 | 0,00 | 100,00 | 26,30 | 73,70 |
| PVC | 21,87 | 78,13 | 0,00 | 100,00 | 46,56 | 53,44 |
